# The strength of neural entrainment to electronic music correlates with proxies of altered states of consciousness

**DOI:** 10.3389/fnhum.2025.1574836

**Published:** 2025-04-09

**Authors:** Raquel Aparicio-Terrés, Samantha López-Mochales, Margarita Díaz-Andreu, Carles Escera

**Affiliations:** ^1^Brainlab – Grup de Recerca en Neurociència Cognitiva, Departament de Psicologia Clínica i Psicobiologia, Facultat de Psicologia, Universitat de Barcelona (UB), Barcelona, Spain; ^2^Institut de Neurociències, Universitat de Barcelona (UB), Barcelona, Spain; ^3^Departament d’Història i Arqueologia, Universitat de Barcelona (UB), Barcelona, Spain; ^4^Institució Catalana de Recerca i Estudis Avançats (ICREA), Barcelona, Spain; ^5^Institut d’Arqueologia de la Universitat de Barcelona (IAUB), Barcelona, Spain; ^6^Institut de Recerca Sant Joan de Déu (IRSJD), Esplugues de Llobregat, Spain

**Keywords:** altered states of consciousness, entrainment, electroencephalography, electronic music, absorption, frequency-tagging

## Abstract

In electronic music events, the driving four-on-the-floor music appears pivotal for inducing altered states of consciousness (ASCs). While various physiological mechanisms link repetitive auditory stimuli to ASCs, entrainment—a brainwave synchronization through periodic external stimuli—has garnered primary focus. However, there are no studies systematically exploring the relationship between entrainment and ASCs. In the present study, we depart from the finding that entrainment to auditory stimuli peaks for stimulation rates around 2 Hz compared to others. Nineteen participants listened to six one-minute electronic music excerpts at different tempos (1.65 Hz, 2.25 Hz, and 2.85 Hz). For each excerpt, they performed cognitive tasks and reported phenomenological experiences related to ASCs through questionnaires. Brain activity was recorded with electroencephalography to assess whether a modulation in entrainment by the beat of electronic music affected objective and subjective proxies of ASCs. Our results revealed a tempo-driven modulation of entrainment at the group level, with entrainment being higher for stimulation rates at 1.65 Hz compared to 2.85 Hz. Similarly, music at 1.65 Hz aroused more feelings of unity compared to music at 2.85 Hz. However, at the individual level, no significant relationship was found between entrainment magnitude and phenomenological experience. Instead, a positive relationship was observed between entrainment and participants’ reaction time. The results suggest that brainwave entrainment modulate processes relevant to rhythm-induced ASCs. While we cannot determine whether participants entered an ASC due to design constraints, the observed relationship between entrainment and reaction time at the individual level supports its functional significance.

## 1 Introduction

Every summer since 2005, thousands of people from around the world gather in Bloom (Flanders, Belgium) to join the Tomorrow land festival. Attendees to this type of electronic dance music events (EDMEs) share the purpose of engaging in transformational experiences in the context of a diversity-friendly environment (Tramacchi, 2000; St John, 2015). The dynamics of these events seem to facilitate their goal (St John, 2015): the dancing, drumming-like music, sleep deprivation and drug consumption (i.e., the 4D’s) that characterize EDMEs are key elements to produce altered states of consciousness (ASCs) (Newson et al., 2021). ASCs might enhance the feelings of connectedness, community, and sacrality that EDMEs’ attendees crave (Tramacchi, 2000). However, the physiological mechanisms by which some of the 4D’s facilitate a transition into an ASC remain underexplored, being most of the research focused on recreational drugs, especially psychedelics (Hood, 2014).

While dancing has been described as religious by its very nature (Gauthier, 2004) and can lead to a loss of self through ‘flow’ states (Csikszentmihalyi, 2014), its physiological effects are increasingly understood, including its role in endorphin release and social bonding (Tarr et al., 2016; Sullivan and Blacker, 2017; Fink et al., 2021). Likewise, sleep deprivation is well-documented to alter brain function (Krause et al., 2017; Zhang et al., 2024) and is related to experiencing dissociative states (Selvi et al., 2015; Creatura et al., 2022). However, the physiological mechanisms underlying how drumming alone contributes to ASC remain less explored (Aparicio-Terrés et al., submitted), despite its ubiquitous role in collective trance experiences across cultures. Given this research gap, we aim to explore a potential cerebral mechanism involved in the 4D’s “drumming,” with a focus on neural entrainment as a possible contributing factor.

The use of repetitive stimuli to induce ASCs is a common practice across cultures and historical periods (Harner, 1990; Krippner, 2000; Díaz-Andreu et al., 2024). This widespread occurrence suggests an underlying biological basis that accounts for this phenomenon (Roberts et al., 2018; Hanslmayr et al., 2019). Neural entrainment has received main attention to explain the link between repetitive stimuli and ASCs (Vaitl et al., 2005). It refers to the synchronization of brainwaves through periodic sensory stimuli (Large, 2008), and seems a consequence of an active brain mechanism to ensure a fine processing of the stimuli (Lakatos et al., 2008; Bittman, 2021).

Cognitive processes can be influenced by exposure to certain frequencies through sensory (Roberts et al., 2018; Hanslmayr et al., 2019) or magnetic (Vernet et al., 2019) stimulation. Therefore, the salient and repetitive beat of electronic music in EDMEs might entrain several brain regions to slow frequencies that are not optimal for some cognitive operations. At the behavioral level, such a physiological configuration would translate into a temporary ASC. However, this brain-state of the mind relationship has never been experimentally measured. In this study, we aim to address this previously unaccounted association.

Studies exploring rhythm-induced ASCs frequently lack measurements of the behavioral correlates associated with these states (Oohashi et al., 2002; Hove et al., 2016; Kawai et al., 2017; Gosseries et al., 2020), leading to descriptive physiological data. However, established tools exist to explore the invariant phenomenological dimensions of ASCs, such as those measured with the 11D-ASC questionnaire (Studerus et al., 2010). Moreover, the cognitive aspects of ASCs can be measured with traditional cognitive tasks used in psychological research.

The present study directly tackles for the first time the assumed relationship between neural entrainment and rhythm-induced ASCs. To this aim, we draw upon the finding that neural entrainment to drum sounds and clicks reaches a peak at around 2 Hz compared to other stimulation rates (Will and Berg, 2007). This physiological property of the brain has never been used to explore how the strength of entrainment affects or is related to different aspects of human functioning and experience. Specifically, we measured (1) participants’ entrainment to the beat of naturalistic electronic music at different tempos with electroencephalography (EEG), (2) objective measures of cognitive function after listening to the electronic music, namely executive function and absorption to the music at different tempos, and (3) participants’ subjective experience during the music listening with three subscales of the 11D-ASC–Experience of unity, Spiritual experience, and Disembodiment. These objective and subjective measures were employed as proxies to ASCs.

The subscales of Experience of unity, Spiritual experience, and Disembodiment were chosen over others because we believe they best capture key phenomenological aspects of rhythm-induced ASCs. As discussed in Newson et al. (2021), feelings of unity are facilitated by synchrony (Tarr et al., 2014, 2016; Lang et al., 2017), a potentially key aspect in inducing awe, which can be measured with the subscale of Spiritual experience. Electronic music contains clear synchronous elements (e.g., in terms of sensorimotor synchronization), and while our participants did not engage in overt synchronous movement, rhythm perception alone may still contribute to these effects. Finally, we selected Disembodiment as it reflects disruptions in bodily self-perception, a phenomenon previously reported in drumming-induced ASCs (Szabó, 2006).

## 2 Methods

The study was approved by the Bioethics Committee of the University of Barcelona and all provisions of the Declaration of Helsinki were followed.

### 2.1 Participants

A total of 20 naïve, healthy volunteers were recruited from online advertisements at the University of Barcelona (Spain). All participants provided written informed consent and were paid for their participation. The general inclusion criteria comprised no history of auditory, neurological or psychiatric disorders and age between 18 and 35 years. One participant was excluded for being unable to comply with the task instructions, resulting in a final sample of 19 participants of ages ranging from 18 to 22 years (2 male and 17 female, *M_age_* = 20). To control for previous musical training, participants filled a standard musical questionnaire used in our research to ascertain both the presence and duration of formal musical training. Out of the 19 participants, six individuals reported prior formal musical education, with a mean duration of 5.833 years (*SD* = 5.269).

### 2.2 Stimuli

**Figure 1:**
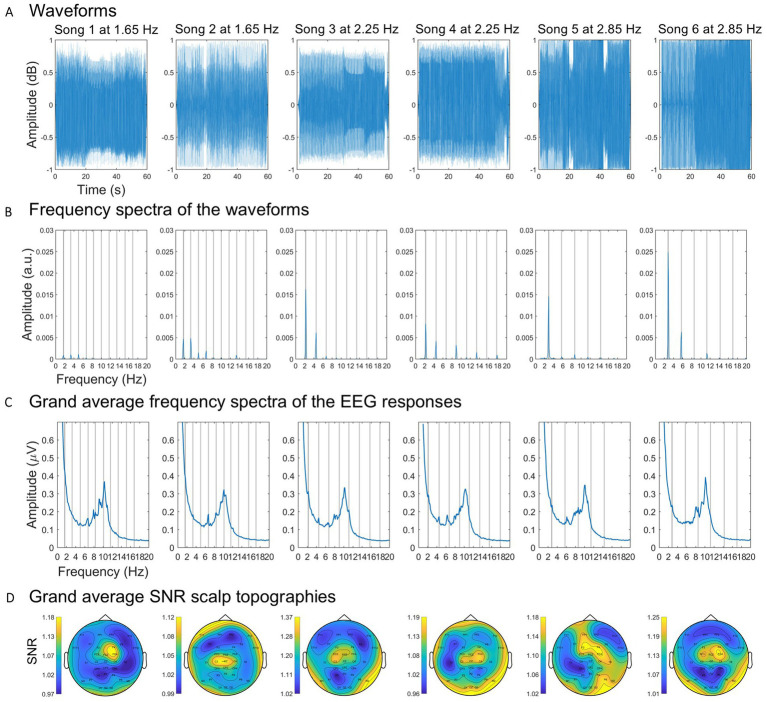
Sound patterns and frequency and spatial representations of the neural responses. **(A)** Waveforms of each song. **(B)** Spectral amplitude of the waveforms. **(C)** Spectral amplitude of the grand average EEG responses to each song. Gray vertical lines represent the beat frequency and harmonics lower than 20 Hz. **(D)** Scalp topographies of the grand average multi-harmonic SNRs.

One-minute-long extracts from “Endless Horizons” by Dhamika (song 1), “El despertar de Joel” by Lab’s Cloud (song 2), “Mind Expander” by Audiomatic (song 3), “Audioslave” by Vertex (song 4), “Check this shit out” by AB (song 5), and “Adagio” by K Complex—Rave Remix (song 6) were used. These extracts were carefully selected to include none to minimal vocals and none to minimal beat drops (see Figure 1A). Each song was modified with v.3.0.0 of Audacity^®^ recording and editing software to include a fade-in and fade-out effect on the first and last seconds, respectively. The approximate tempos of the six songs were determined in a semi-automatic approach (see Section 2.4). The tempos were: 1.65 Hz or 99 bpm (song 1 and 2), 2.25 Hz or 135 bpm (song 3 and 4), and 2.85 Hz or 171 bpm (song 5 and 6). The six songs could therefore be classified into three tempos: 1.65 Hz, 2.25 Hz and 2.85 Hz. The sound intensity levels were intentionally not equalized across the song extracts because doing so would not effectively address the natural differences in the volume of the beat among the different songs. However, all the extracts were played at the same intensity level.

### 2.3 Procedure

**Figure 2:**
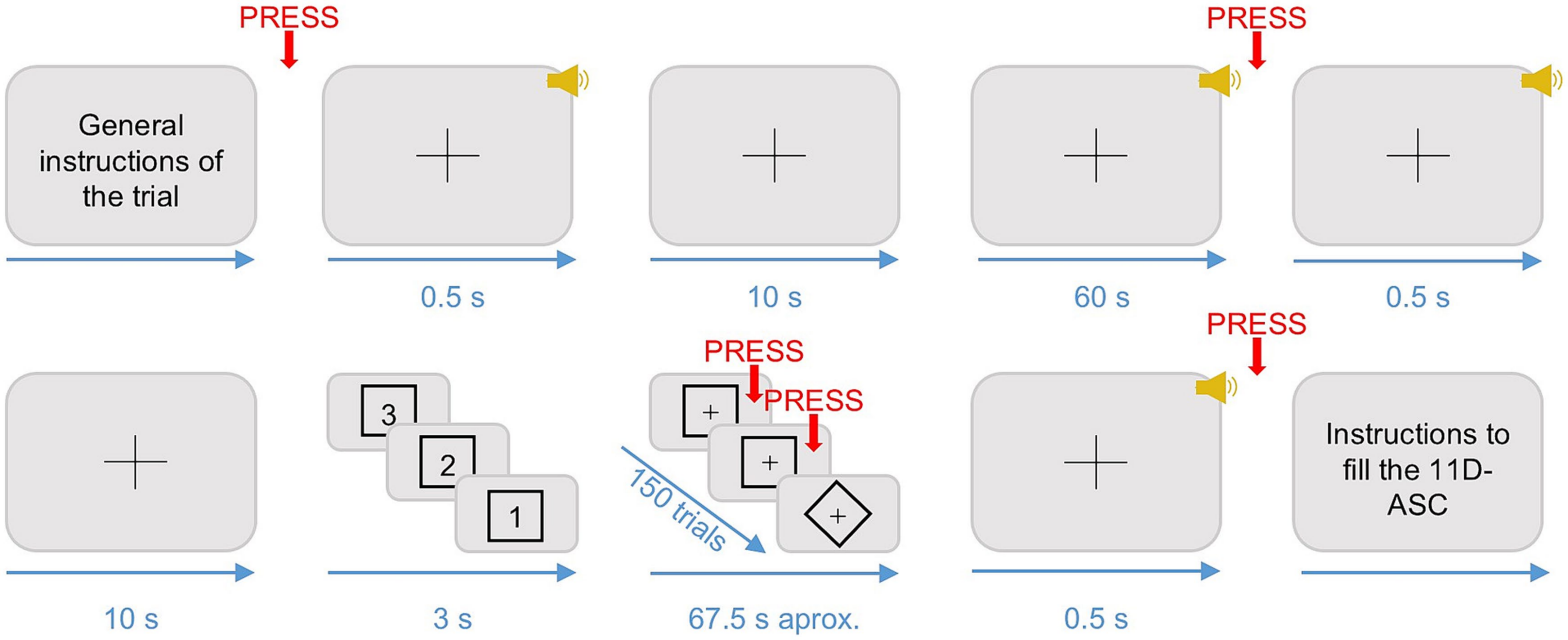
Schematic representation of the experimental design. The figure shows the sequence of motor responses (in red), auditory stimuli (in yellow), and visual stimuli (in black) that participants produced or were presented with during each trial. The black crosses represent fixation crosses, which were present throughout the entire duration of the trials. The timing constraints (in blue) are represented in seconds. Adapted from Studerus et al. (2010).

Participants sat comfortably in a soundproof Faraday chamber with a screen in front of them, headphones on, a mouse in their dominant hand, and a touch sensitive tablet on their legs. A graphical representation of the sequence of an example trial, including the visual stimuli presented to participants, can be seen in Figure 2.

**Figure 3:**
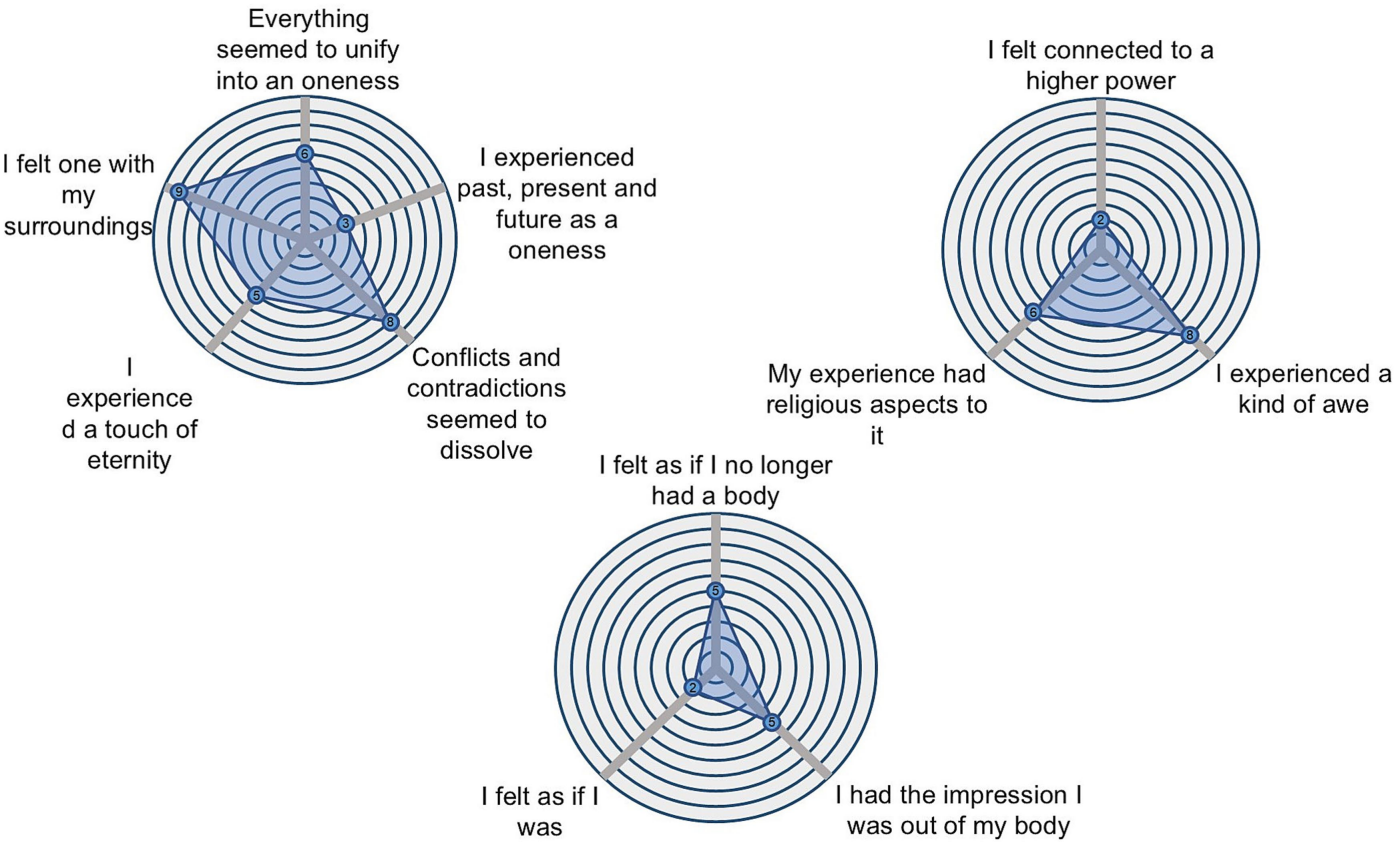
Customizable spider charts used to obtain ratings for the subscales of the 11D-ASC questionnaire. Each spider chart represents a subscale, with as many radii as items. The spider charts were displayed on a touch-sensitive tablet, where participants filled in the subscales by drawing their finger.

At the beginning of each trial, participants were presented with a 0.5-s-long pure tone indicating the start of a 10 s silence period. Immediately after, one of the six one-minute-long songs was played at 80 dBs. Participants were instructed to listen to the music, maintain their eyes open, gaze fixed on the fixation cross displayed on the screen, and to move as little as possible during the music listening. In total, there were three trials for each song randomized across three blocks for each participant. When the music stopped, participants were instructed to left click on the mouse as soon as they realized the music was over. After 10 s, a 0.5-s-long pure tone warned participants that a go/no-go task was about to start. Then, the screen displayed a countdown starting from three and framed by a visual shape that could either be a square or a diamond shape. Once the countdown reached one, 150 shapes (either a square or a diamond) were presented sequentially in the screen, following the same timing constraints as in a previous study (Nilsen et al., 2020). Specifically, prior to the presentation of each shape, a fixation cross was presented for 200 ms + 0–300 ms jitter. Following the fixation cross, each shape was displayed up to 700 ms or until response. If the shape in the screen matched the one presented during the countdown, participants had to left click on the mouse (go stimuli). If not, they had to refrain from clicking (no-go stimuli). The shape of the go-stimuli was randomized for each participant. The probability of a go-stimuli appearing was fixed in each trial to 75% to maximize the number of false alarms (i.e., responding when a no-go stimulus is presented) (Young et al., 2018). When the go/no-go task was over, participants used the touch-sensitive tablet to fill from 0 to 10 the items presented in a spider chart of three subscales of the 11D-ASC (Studerus et al., 2010), namely Experience of unity, Spiritual experience, and Disembodiment. The ratings were obtained using three customable spider charts, one for each subscale, with as many radii as items (Figure 3) (López-Mochales et al., 2023). A fixation cross in the center of the screen was present during the whole duration of the trials.

Prior to the start of the experiment, all participants conducted three practice trials, in which generic elevator music was used as stimuli, to get familiarized with the tasks. EEG was recorded during the whole duration of the trials.

### 2.4 Sound pattern analysis

The sound pattern analysis was conducted with the Fieldtrip v.20211020 toolbox (Oostenveld et al., 2011) and Matlab v.2021a. The frequency at which entrainment was expected to be elicited in the recorded EEG signals (i.e., the songs’ beat frequency) was determined in a semi-automatic approach. First, an experienced musician tapped along to each 1-min song extract on a keyboard, while a custom Matlab program counted the taps and extracted a first approximated beat frequency (i.e., the number of taps per second). The second step involved transforming the songs’ waveforms into the frequency dimension. To this aim, each song was cut from +1 s to +59 s relative to the onset of the musical extract. The first and last seconds of the songs were left out of the analysis to exclude the fade-in and fade-out effects of the songs. The envelopes were extracted using a Hilbert transform, this is, by obtaining the absolute value of the complex-valued analytic signal using the functions ‘abs’ and ‘hilbert’ in Matlab. Considering the non-stationarities inherent in our naturalistic music stimulation, the envelopes were transformed into the frequency domain using Welch’s method with a window length of 10 s and a 90% overlap, employing a Hanning taper. This transformation was performed using the ‘mtmconvolv’ function implemented in Fieldtrip and resulted in a frequency spectrum with a frequency resolution of 0.1 Hz. The exact beat frequency was determined as the spectral amplitude’s peak within a range from −0.5 to +0.5 Hz from the first approximated beat frequency.

To explore the temporal dynamics of neural entrainment and to measure the strength of entrainment at the last seconds of stimulation a similar procedure was followed. The exact beat frequency was measured across time in a separate analysis. The 58-s-long waveforms were segmented into 10 s windows with a 90% overlap and subsequently transformed into the frequency domain with a Hanning taper, as implemented in Fieldtrip’s function ‘mtmconvolv’, yielding frequency spectra with a frequency resolution of 0.1 Hz. The exact beat frequency for each window was determined as the frequency bin with the maximum spectral amplitude within a range from −0.5 to +0.5 Hz from the approximated beat frequency.

Differences in the spectral amplitude of the beat frequencies between songs were expected and indeed confirmed after the frequency transformations (Figure 1B). These differences might be explained by there being a different number of beats for each musical extract, which might affect the measures of neural entrainment in an unknown way. To account for these differences, we implemented a normalization procedure. Global entrainment and entrainment across time were measured as a multi-harmonic signal-to-noise ratio (SNR) of the spectral amplitudes of the EEG responses at the beat and significant harmonic frequencies (see section 2.6. EEG data processing and analysis). To normalize these measures, we quantified for each song the multi-harmonic SNR of the spectral amplitudes of the whole song waveforms at the beat and significant harmonic frequencies. Subsequently, we computed the ratio between the SNR of the EEG responses and the SNR of the corresponding waveforms. To compute the multi-harmonic SNR of the waveforms, we first summed the spectral amplitudes at the beat frequency (F0), first harmonic (2F0), and second harmonic (3F0) frequency bins (i.e., signal) and at the corresponding neighbor frequencies with one frequency bin of spacing at each flank (i.e., baseline). The multi-harmonic SNR of the waveforms were computed by dividing the signal’s amplitude by the baseline’s amplitude.

### 2.5 EEG recordings

EEG was continuously recorded during the whole duration of the trials and digitalized at a sampling rate of 1 kHz by a Neuroscan SynAmps RT amplifier (Neuroscan, Compumedics, Charlotte, NC, USA). For the EEG acquisition, 36 sintered electrodes mounted in a neoprene cap (Quick-Cap Neo-Net; Compumedics, Charlotte, NC, USA) at standard locations according to the extended 10–10 system were used. Electrooculograms (EOG) were measured with two bipolar in-cap electrodes placed above and below the right eye (VEOG), and two horizontal in-cap electrodes placed on the outer canthi of the eyes (HEOG). The ground electrode was located at AFz and the common reference electrode between Cz and Cpz. All impedances were kept below 10 kΩ during the whole recording session.

### 2.6 EEG data processing

The data analysis was performed offline using the EEGlab v.2021.1 toolbox (Delorme and Makeig, 2004) and the Fieldtrip v.20211020 toolbox (Oostenveld et al., 2011) running under Matlab v.2021a. For each participant, the continuous recordings were filtered from 0.5 Hz to 45 Hz with a Finite Impulse Response bandpass Kaiser filter to remove slow drifts and line noise, respectively. Excluding the bipolar montages, the filtered data were re-referenced to the average activity of all electrodes to remove any noise from the reference electrode. For each trial, the continuous recordings were segmented from −10 s to +60 s relative to the onset of the auditory stimuli. All epochs were merged to detect muscle artifacts based on a semi-automatic approach. First, all epochs were visually inspected with respect to their waveform morphology. Those epochs that included muscle artifacts (Goncharova et al., 2003), characterized by high-frequency activity (> 20 Hz) and typically produced by muscular activity near the head, such as swallowing or moving the head, were rejected, unless the artifacts were due to eye movements. Then, independent component analysis (ICA) was computed to remove artifacts produced by eye blinks, eye movements, and heart activity from the EEG signal using the runica algorithm (Bell and Sejnowski, 1995; Makeig et al., 1997). Deliberately removing activity in the EEG coming from the heart was critical because the tempo of the songs at 1.65 Hz partly matched the frequency of the human heart, ranging from 1 Hz to 1.67 Hz. Lastly, epochs were baseline corrected by using the EEG activity over the 10 s windows prior to the onset of the songs. The electrodes relative to the vertical and horizontal EOGs were not further included in the analyses.

For each participant and trial, epochs lasting 58 s were sorted by segmenting the pre-processed recordings from +1 to +59 s relative to the onset of the auditory stimuli. Following the same procedure as in a previous study (Nozaradan et al., 2012a), the first second of each epoch was removed: (1) to discard the transient auditory evoked potentials related to the onset of the stimulus (Saupe et al., 2009; Nozaradan et al., 2011, 2012b); (2) because steady-state evoke potentials require several cycles of stimulation to be steadily entrained (Regan, 1989); and (3) because several repetitions of the beat are required to elicit a steady perception of beat (Repp, 2005). Additionally, by removing the first and last seconds of the recording, the fade-in and fade-out effects of the stimuli were excluded.

### 2.7 EEG data analysis

After pre-processing, EEG data were analyzed in two independent strategies. The first strategy involved calculating *global* measures of entrainment by computing the SNR at the frequency beats and harmonics across the entire duration of the stimulation. The second strategy focused on *time-resolved entrainment*, where the SNR at the frequency beat and harmonics was computed using a sliding window approach with 10-s-long segments of data.

To explore *global* measures of entrainment, the obtained 58-s-long epochs were transformed into the frequency domain by using Welch’s method, computed over 10 s windows with a 90% overlap with a Hanning taper, as implemented in Fieldtrip’s function ‘mtmconvolv’ (Figure 1C). This procedure yielded frequency spectra with a 0.1 Hz frequency resolution. For each participant, song and electrode, the resulting frequency spectra were averaged across trials. Global entrainment to each song was measured as a multi-harmonic SNR response, meaning that the measure of entrainment was not only limited to the spectral amplitude at the beat frequency. Rather, entrainment was assessed as a multi-harmonic response due to the non-sinusoidal beats of the songs and the nonlinear nature of the brain processes in response to acoustic onsets that might project the neural responses to the beat frequency onto higher harmonics of the beat frequency (Zhou et al., 2016; Retter et al., 2021).

To determine which harmonics to consider, the significance of each harmonic’s spectral amplitude averaged across participants and electrodes was tested for each song. Specifically, we z-scored the group-level spectral amplitude at the frequency of each harmonic (i.e., signal), with a baseline defined as the corresponding neighboring bins with one frequency bin of spacing, using the formula z (signal) = (signal − baseline mean)/baseline SD. This testing process was carried out sequentially for each harmonic until one harmonic did not reach significance (Lenc et al., 2018). Using this test, the first and second harmonics were considered significant for songs 5 and 6, as they had z-scores >1.64 (i.e., *p* < 0.05, one-sample, one-tailed test; testing signal > noise). To prevent introducing bias into the results, the same harmonics were employed for computing entrainment across all songs, namely the first and second harmonics. For each participant, song, and electrode we summed the spectral amplitudes at the beat, first harmonic, and second harmonic frequency bins (i.e., signal) and at the baseline bins (Retter et al., 2021). Lastly, we computed the SNR between the signal and averaged baseline spectral amplitudes.

Considering previous findings indicating that the perception of beat is region-specific (Grahn and Brett, 2007; Chen et al., 2008), we examined the scalp-wide distribution of entrainment to identify and select electrodes that are pertinent to the observed entrainment patterns. To this aim, for each song and electrode we averaged the SNR measures across participants and plotted the scalp distribution (Figure 1D). Upon a visual examination, a consistent fronto-central pattern of entrainment across songs was observed. Therefore, for each participant and song, we quantified global entrainment as a multi-harmonic SNR averaged across fronto-central channels (i.e., FC3, FCz, FC4, C3, Cz, and C4 according to the extended 10–10 standard system). These measures were subsequently normalized by the spectral amplitudes of the songs (see section 2.4. Sound pattern analysis). Lastly, to mitigate potential confound effects related to the idiosyncratic spectral characteristics of the songs, global entrainment was averaged for each participant between songs within tempos.

The second analytical strategy was related to exploring the *temporal dynamics* of neural entrainment for each participant and song. To this aim, the 58-s-long average epochs were split into 10 s windows with a 90% overlap and subsequently transformed into the frequency domain with a Hanning taper, as implemented in Fieldtrip’s function ‘mtmconvolv’. For each participant, song, electrode and window, the resulting frequency spectra were averaged across trials, yielding frequency spectra with a frequency resolution of 0.1 Hz. The deliberate decision of computing FFTs given windows of 10 s is justified by previous literature on entrainment using similar short-lasting epochs (Okawa et al., 2017; Tal et al., 2017). Also, using overlapping sliding time windows is justified by the fact that we are conducting a fine-grained analysis of entrainment at specific frequencies (Batterink and Choi, 2021). The significant harmonics for computing entrainment as a multi-harmonic SNR response across time were determined to be the same as in the measurement of global entrainment, namely the first and second harmonics. For each participant, song, electrode and window we summed the spectral amplitudes at the beat, first harmonic, and second harmonic frequency bins (i.e., signal) and baseline bins (Retter et al., 2021) and computed the SNR. Subsequently, entrainment across time was quantified as the multi-harmonic SNR averaged across fronto-central channels (i.e., FC3, FCz, FC4, C3, Cz, and C4 according to the extended 10–10 standard system). These measures were normalized by the spectral amplitudes of the songs (see section 2.4. Sound pattern analysis). Lastly, entrainment was averaged for each participant and window between songs within tempos.

### 2.8 Analysis of behavioral measures

#### 2.8.1 Reaction time task

Reaction time (RT) was measured as the time in seconds that participants took to respond to the offset of the songs. For each participant, RT was averaged across trials within songs to control for random variability in their performance. For each tempo, RT was averaged between songs to control for possible confound effects provoked by the idiosyncratic musical characteristics of the songs.

#### 2.8.2 Go/no-go task

Participants’ responses in the go/no-go task were classified in four types: (a) hits (HTs), if participants pressed the mouse when a go-stimulus was presented; (b) misses (MSs), if participants did not press the mouse when a go-stimulus was presented; (c) correct rejections (CRs), if participants did not press the mouse when a no-go stimulus was presented; and (d) false alarms (FAs), if participants pressed the mouse when a no-go stimulus was presented. For each participant and trial, the proportion of each type of response was calculated by dividing the total number of those responses by the total number of trials within the go/no-go task. The proportions for each type of response were averaged across trials within the same song, and the mean punctuation for each type of response was obtained by calculating the mean across songs within each tempo.

#### 2.8.3 11D-ASC

For each participant, trial and subscale (i.e., Disembodiment, Spiritual experience, and Experience of unity), the average score was computed across items. For each participant, the punctuations in each subscale were averaged across trials within songs. For each tempo, the scores in each subscale were averaged between songs.

### 2.9 Statistical methods

To evaluate group-level differences across tempo conditions in neural entrainment, performance in cognitive tasks, and phenomenological experience we employed a series of statistical analyses. First, to assess differences in global neural entrainment across tempo conditions, we fitted a linear mixed model. “Musical training” (indicating whether participants had any form of musical training or not) and “years of musical training” (representing the duration of musical training in years), as measured with the musical questionnaire, were added as covariates to control for potential influences of musical expertise. A random effect for each participant’s identification code was included to account for within-subject correlations across repeated measurements. Model selection was guided by changes in the Bayesian Information Criterion (BIC), with the best-fitting model determined based on significant reductions in BIC. Post-hoc comparisons were then conducted using Tukey’s tests with Kenward-Roger degrees of freedom to identify significant differences between tempo conditions. Second, to examine the temporal dynamics of entrainment, an Analysis of Variance (ANOVA) was performed at each sliding time window. When parametric assumptions were violated, non-parametric alternatives, specifically the Kruskal-Wallis test, were employed. Bonferroni corrections were applied to control for multiple comparisons, and a stringent significance threshold of *p* < 0.01 was set to mitigate type I error inflation. *Post hoc* comparisons were conducted exclusively at the final time point of entrainment measurement, as this served as the basis for correlational analyses between entrainment and cognitive measures. Lastly, behavioral differences across tempo conditions were analyzed using one-way repeated measures ANOVAs for reaction time, executive function, and phenomenological experience subscales. In cases of non-normally distributed data, we conducted Friedman tests as a non-parametric alternative.

To explore brain-behavior relationships, we examined the association between neural entrainment and both objective (i.e., reaction time and executive function) and subjective measures (i.e., scores on Experience of Unity, Spiritual Experience, and Disembodiment) by fitting linear models. The significance and strength of the relationships were assessed via the slope of the linear models (i.e., beta coefficients) and R^2^ values. In these analyses, the response variable was the difference score of the respective measure across pairs of tempo conditions, while the predictor variable was the difference score in neural entrainment across the same tempo pairs. Correlating difference scores aimed to determine whether variations in neural synchronization to the beat—ranging from high to low—were associated with fluctuations in participants’ performance between the two conditions. Additionally, for objective measures, we used neural entrainment from the final 10-s window, as this period was hypothesized to exert the strongest influence on subsequent cognitive task performance. For subjective measures, we used global neural entrainment, as phenomenological experiences reported in a retrospective questionnaire were expected to be influenced by the overall course of stimulation rather than moment-to-moment fluctuations.

## 3 Results

### 3.1 Entrainment

#### 3.1.1 Global entrainment

A linear mixed model analysis was conducted by using the ‘lmer’ function from the lme4 package (Bates et al., 2014) in Rstudio v.4.1.3 to explore the effect of tempo on global entrainment. The covariates “musical training” and “years of musical training” were included in the analysis to control for any potential influence of participants’ musical expertise on entrainment. Each participant’s identification code was added as a random effect to account for the correlation among the repeated measurements from the same individual. The normality and dispersion of the residuals were checked. Adding the effect of tempo and the covariates related to musical training improved the fit of the model, as evidenced by a significant decrease of the Bayesian information criterion (BIC) in the model with all the effects (*BIC* = −84.391) compared to the null model (*BIC* = −80.916; *χ^2^*_(4)_ = 19.648, *p* < 0.01) (Jeffreys, 1961; Kass and Raftery, 1995). The results of the model including the main effect of tempo and the covariates related to musical training are shown in Table 1. The compared linear mixed models expressed in Wilkinson-Rogers notation (Wilkinson and Rogers, 1973) are provided in the Supplementary material A1.

**Table 1:** Results of the mixed effect model including the main effect of tempo, musical training and years of musical training.

	Coef β	SE	t	95% CI	*p*
Intercept	1.052	0.023	44.865	[1.008 1.096]	< 0.001 ∗∗∗
Tempo 2.25 Hz	−0.065	0.030	−2.182	[−0.123–0.007]	0.036 ∗
Tempo 2.85 Hz	−0.106	0.030	−3.546	[−0.164–0.048]	0.001 ∗∗
Musical Training	−0.040	0.040	−1.007	[−0.116 0.036]	0.329
Years of musical training	0.0128	0.005	2.644	[0.003 0.022]	0.018 ∗∗

Participant identification codes were added as a random effect. Coefficient comparisons for main effects are given as entrainment to 1.65 Hz vs. 2.25 Hz and entrainment to 1.65 Hz vs. 2.85 Hz. ∗ indicates significance level.

**Figure 4:**
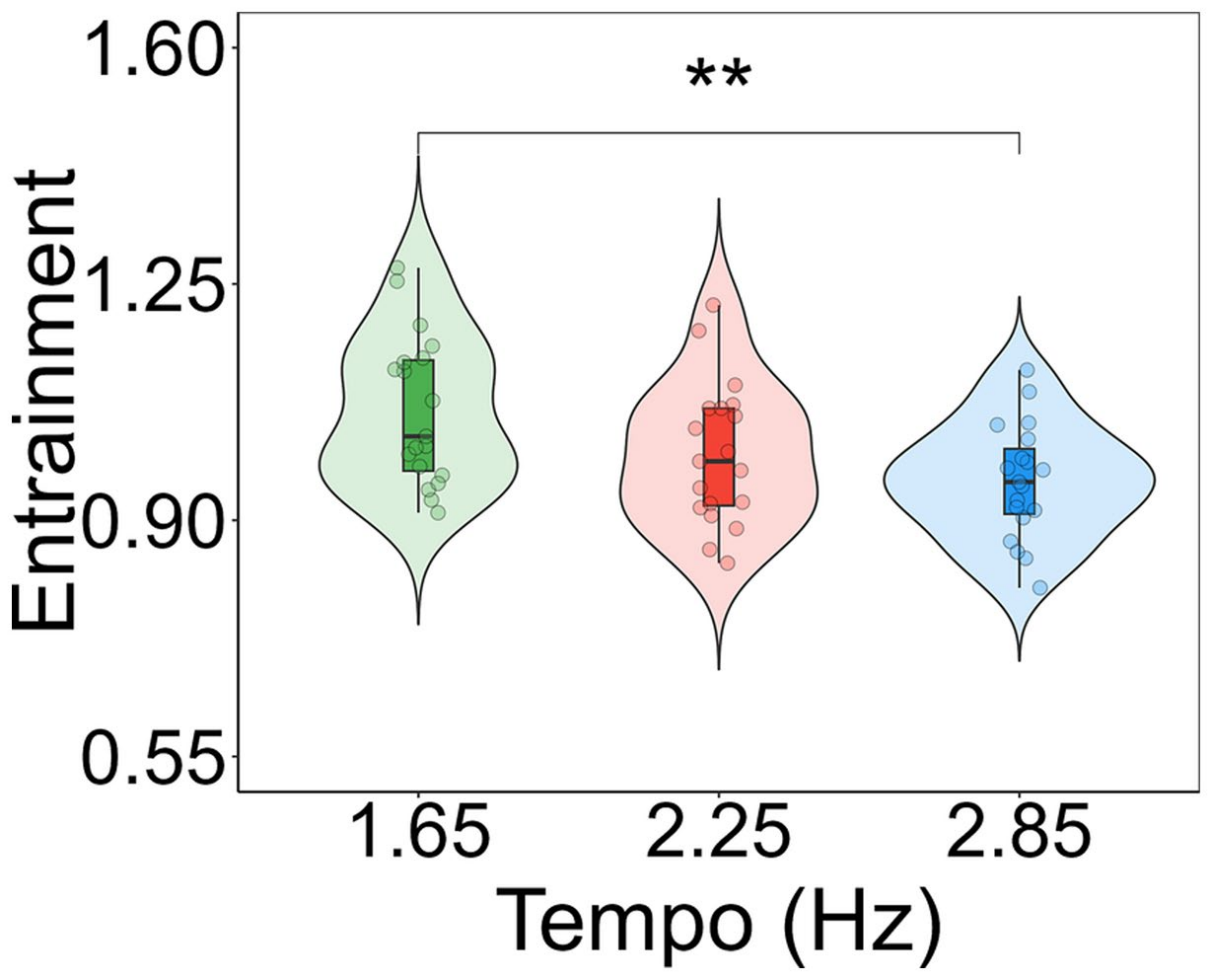
Violin plots showing entrainment to each tempo condition. Entrainment is shown as a multi-harmonic signal-to-noise ratio response normalized by the spectral variations in the musical extracts. Individual data points for each condition are displayed as circular markers. **Indicates a *p*-value lower than 0.01.

To determine the significant comparisons of entrainment between tempos, Tukey’s post-hoc tests with the Kenward-Roger degrees of freedom method were conducted by using the ‘emmeans’ function (Lenth, 2019) in Rstudio v.4.1.3. Post-hoc contrasts revealed that entrainment was significantly greater for 1.65 Hz compared to 2.85 Hz (*t_(36)_* = 3.546, *p* = 0.003; *M_1.65_* = 1.067, *M_2.85_* = 0.990; Figure 4). No other differences between conditions were found.

#### 3.1.2 Entrainment across time

**Figure 5:**
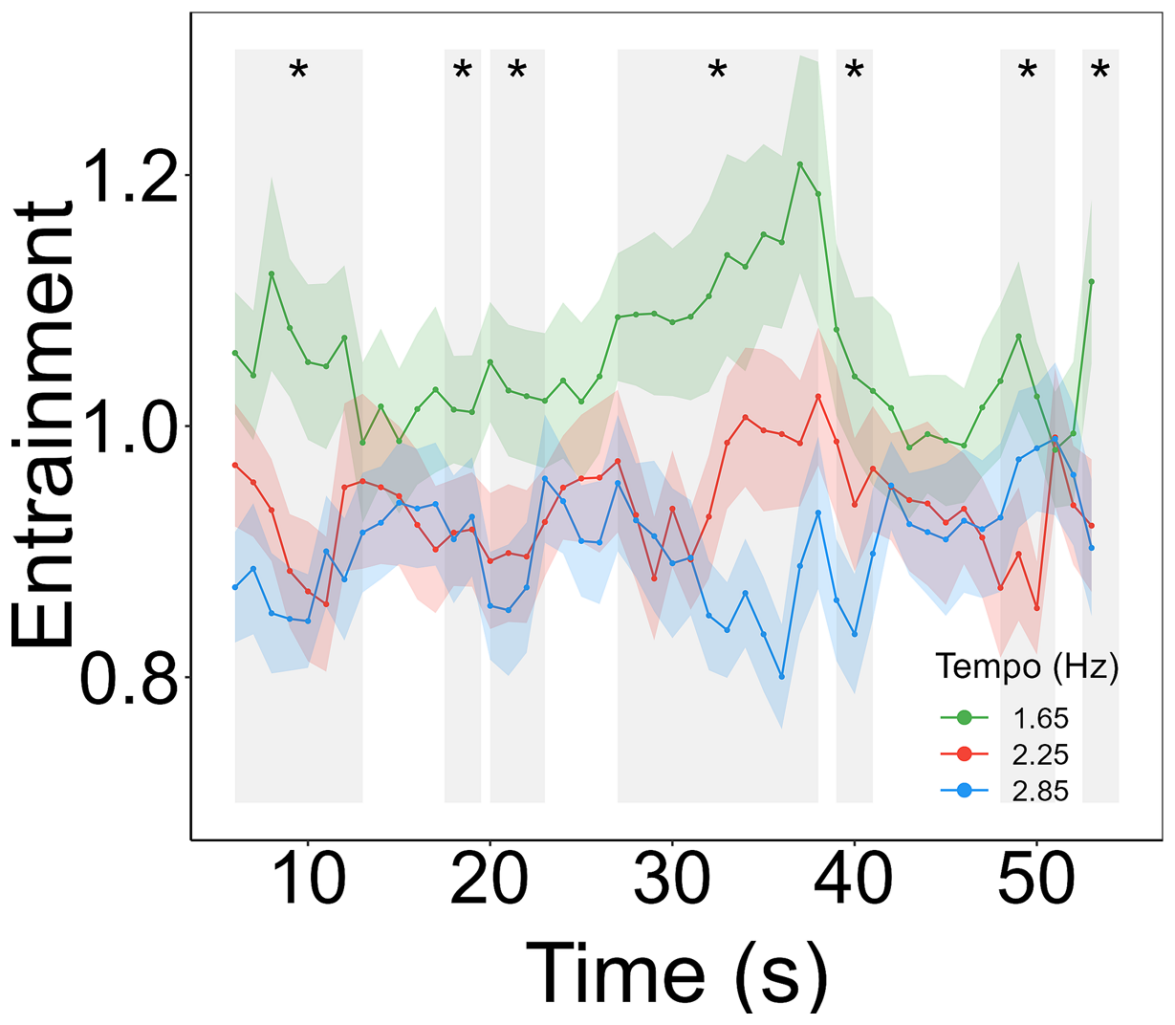
Time-series of entrainment to each tempo condition. Entrainment is shown as a multi-harmonic signal-to-noise ratio response normalized by the spectral variations in the musical extracts. Gray shaded windows represent significant main effects of tempo. *Indicates *p*-value lower than 0.01.

To assess differences in the temporal dynamics of entrainment to the beat of the songs at the different tempos, 48 one-way repeated measures ANOVAs were conducted, one for each sliding window, with one factor (tempo) and three levels (1.65, 2.25, and 2.85 Hz). A significance level of *p* < 0.01 was implemented to account for the multiple ANOVAs and mitigate the risk of type I error. Depending on the window, post-hoc analyses were conducted using *t*-tests for normally distributed data or Wilcoxon signed-rank tests for non-normally distributed data. Bonferroni corrections were applied to account for multiple comparisons. The statistical results are not reported due to the significant quantity of statistical analysis conducted. Figure 5 visually depicts the results of the main effects across all time points. However, the last time point in which entrainment was measured, which served as the basis for correlational analyses between entrainment and RT and executive function, require further examination. Specifically, a significant effect of tempo was found on the last time point (*χ^2^_(2)_* = 9.789, *p* = 0.007, *W* = 0.258). Post-hoc comparison tests revealed that entrainment was higher for songs at 1.65 Hz compared to both 2.25 Hz (*t_(2)_* = 163*, p* = 0.010) and 2.85 Hz (*t_(2)_* = 165*, p* = 0.010; *M_1.65_* = 1.115, *M_2.25_* = 0.920, *M_2.85_* = 0.903).

### 3.2 Behavioral measures

#### 3.2.1 Reaction time

To explore differences in RT between tempos, a non-parametric Friedman test of differences among repeated measures was conducted. The results showed no significant differences in RT between tempos (*χ^2^*_(2)_ = 2, *p* = 0.368, *W* = 0.053; *M_1.65_* = 1.044, *M_2.25_* = 1.208, *M_2.85_* = 1.018).

#### 3.2.2 Go/no-go task

For each of the response types (HT, MS, CR and FA) we performed a one-way non-parametric Friedman test of differences among repeated measures. The results showed no significant differences between tempos across all response types (HT: *χ^2^*_(2)_ = 3.361, *p* = 0.186, *W* = 0.088; *M_1.65_* = 0.723, M*_2.25_* = 0.728, *M_2.85_* = 0.725; MS: *χ^2^*_(2)_ = 3.041, *p* = 0.219, *W* = 0.080; *M_1.65_* = 0.023, *M_2.25_* = 0.019, *M_2.85_* = 0.022; CR: *χ^2^*_(2)_ = 2.842, *p* = 0.241, *W* = 0.074; *M_1.65_* = 0.206, *M_2.25_* = 0.202, *M_2.85_* = 0.2; FA: *χ^2^*_(2)_ = 2.842, *p* = 0.241, *W* = 0.074; *M_1.65_* = 0.048, *M_2.25_* = 0.051, *M_2.85_* = 0.053).

#### 3.2.3 11D-ASC

**Figure 6:**
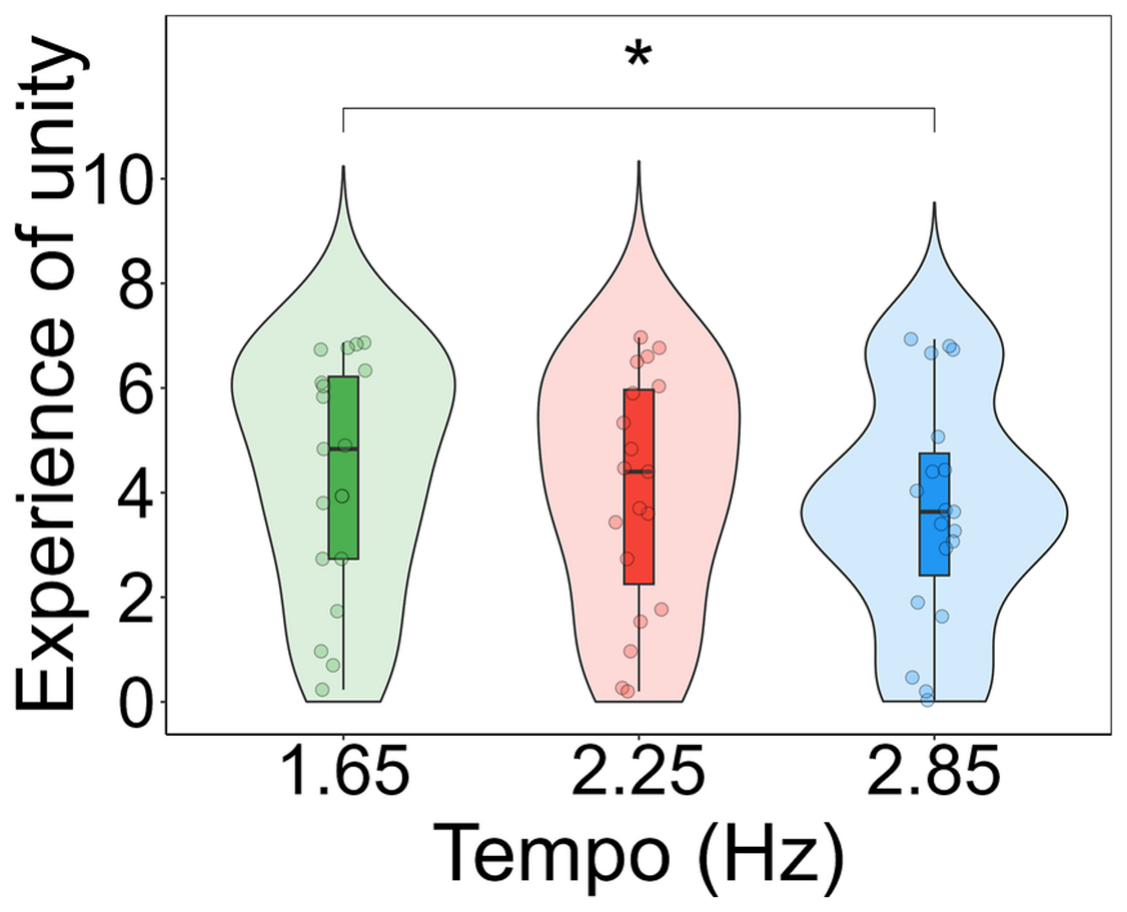
Violin plots showing participants’ scores in the subscale experience of unity for each tempo condition. Individual data points for each condition are displayed as circular markers. *Indicates a *p*-value lower than 0.05.

For each subscale of the 11D-ASC (Disembodiment, Spiritual experience, and Experience of unity) a one-way repeated measures Analysis of Variance was conducted to investigate differences in these scores between tempos. The results showed a significant effect of tempo in Experience of unity (*F*_(2, 36)_ = 4.775, *p* = 0.014, *η^2^* = 0.016). A post-hoc analysis using t-tests revealed a significant difference (*t*_(18)_ = −2.567, *p* = 0.019) between the tempos 1.65 Hz (*M_1.65_* = 4.316) and 2.85 Hz (*M_2.85_* = 3.646; Figure 6). No differences were found in the scores of Disembodiment (*F*_(2, 36)_ = 1.309, *p* = 0.277, *η^2^* = 0.000716; *M_1.65_* = 3.893, *M_2.25_* = 3.444, *M_2.85_* = 3.447) and Spiritual Experience (*F*_(2, 36)_ = 0.214, *p* = 0.808, *η^2^* = 0.008; *M_1.65_* = 2.579, *M_2.25_* = 2.62, *M_2.85_* = 2.687).

### 3.3 Brain-behavior correlation analyses

**Figure 7:**
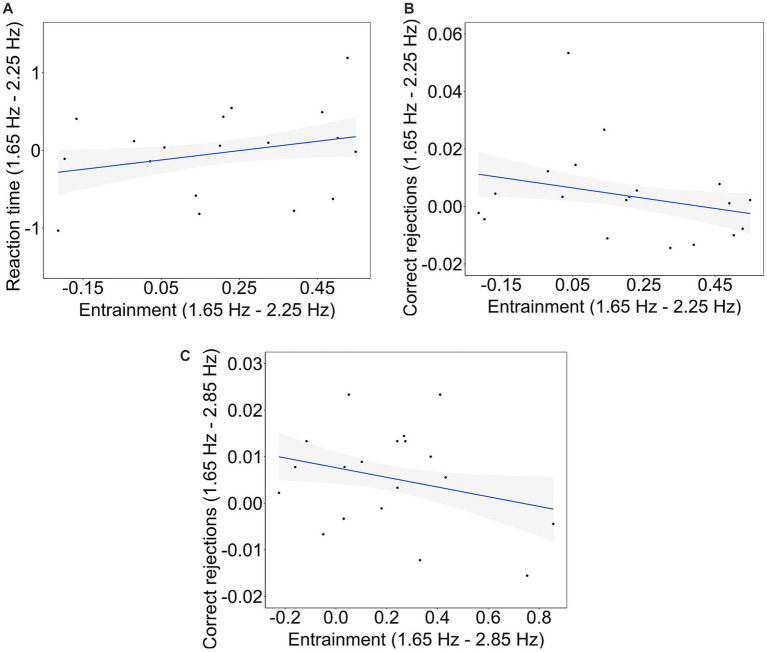
Brain-behavior significant correlations. Relationship between **(A)** the difference in RT and the difference in entrainment between tempos 1.65 Hz and 2.25 Hz, **(B)** the difference in CR and the difference in entrainment between tempos 1.65 Hz and 2.25 Hz, and **(C)** the difference in CR and the difference in entrainment between tempos 1.65 Hz and 2.85 Hz. The regression lines representing the relationships are represented by solid lines, while the shaded areas depict the confidence ranges for each regression at their respective tempos.

To explore whether the magnitude of entrainment is related to participants’ performance in the objective behavioral measures, linear models were applied to the differences in RT, executive function and entrainment between each pair of tempos. Computing the correlations across differences aimed to uncover whether states in which the brain is highly synchronized to the beats of the songs vs. less synchronized is related to the variability in the participant’s performance between the two conditions. The significance of the relationships was assessed by testing the significance of the slopes of the linear models. To this aim, only entrainment calculated from the last 10-s-long windows was used. This is because participants’ performance in cognitive tasks can be expected to be affected by the brain configuration at the closest moment in time to the task. Also, as the percentage of CR and HT, and the percentage of MS and FS are complementary, correlations with entrainment were only computed for correct rejection and miss responses. The normality of the residuals for each linear model was assessed using Shapiro–Wilk’s tests, revealing that all residuals exhibited a normal distribution. The analysis revealed a significant relationship between the differences in RT and entrainment between tempos 1.65 Hz and 2.25 Hz (*B* = 0.942, *t*_(17)_ = 2.276, *p* = 0.027, *R^2^ =* 0.086; Figure 7A). Also, there was a significant relationship between the differences in CR and entrainment between tempos 1.65 Hz and 2.25 Hz (*B* = −0.018, *t_(17)_* = −2.196, *p* = 0.032, *R^2^ =* 0.081; Figure 7B) and between 1.65 Hz and 2.85 Hz (*B* = −0.010, *t_(17)_* = −2.118, *p* = 0.039, *R^2^ =* 0.075; Figure 7C). No other significant relationships were observed.

To investigate whether the magnitude of entrainment is associated with the subjective experience of participants while listening to the songs, linear models were applied to the differences in participants’ scores in the 11D-ASC subscales and the differences in entrainment between each pair of tempos. In contrast to the objective measures, participant-reported measures of their experience during the stimulation can be expected to be influenced by the overall level of entrainment. No significant relationships were found. All the tested linear mixed models expressed in Wilkinson-Rogers notation (Wilkinson and Rogers, 1973) are provided in the Supplementary material A2.

## 4 Discussion

The present study has revealed, using electroencephalography, a relationship between the magnitude of entrainment to the beat of electronic music and some aspects of cognition. We have shown that the strength of entrainment to the beat of 1-min-long electronic music can be modulated by the tempo of the music. Our results show that entrainment is higher for stimulation rates at 1.65 Hz compared to faster rates of stimulation, namely 2.85 Hz, but not in comparison to 2.25 Hz. In examining the temporal dynamics of entrainment, a similar pattern of results is observed throughout the time course of the songs. Notably, towards the end of the songs, entrainment to the stimulation at 1.65 Hz was higher compared to the other two beat frequencies. The observed neural differences between conditions allowed us to explore whether the magnitude of entrainment is related to differences in cognitive processes or subjective experiences related to altered states of consciousness (ASCs). Although the participants’ reaction time, executive function, feelings of disembodiment, and spiritual experiences were not different depending on the tempo of the music they were exposed to, it was found that music at 1.65 Hz aroused more feelings of unity compared to music at 2.85 Hz. At the level of individual participants, we did not observe any significant relationship between the magnitude of entrainment and participants’ phenomenological experiences during listening to the songs. However, our findings revealed significant yet weak relationships between the magnitude of entrainment and both participants’ reaction time and response inhibition.

Previous empirical evidence showed that entrainment is maximum for periodic auditory stimuli at a rate of 2 Hz within a range of frequencies from 1 to 10 Hz in steps of 1 Hz (Will and Berg, 2007). Our results align with and build upon these findings, having employed stimulus frequencies within a range from 1 to 3 Hz. Specifically, we found more entrainment for music with a beat frequency closer to 2 Hz, namely at 1.65 Hz, compared to music closer to 3 Hz, this is, at 2.85 Hz. We observed that this pattern remains consistent when considering the temporal dynamics of entrainment over the time course of the 60-s musical excerpts. Nevertheless, it is important to consider the large fluctuations in entrainment across time (Figure 5). These fluctuations could be potentially attributed to stimulus properties, namely variations in the beat accent in the electronic music. However, we contend against this notion due to two main reasons. First, the presence of the beats within the excerpts are quite consistent across time, as visually depicted in Figure 1A. Second, two songs were added for each tempo to lower the effect of the idiosyncratic musical characteristics of each song on the measures of entrainment, attenuating the influence of beat-related stimulus properties. It remains unclear why entrainment to beat frequencies of 2.25 Hz, which is closer to 2 Hz compared to 1.65 Hz, do not exhibit the highest level of entrainment across the tempos used in the study.

The predominantly fronto-central scalp distribution of entrainment also aligns with previous findings (Tierney and Kraus, 2015). This pattern can be attributed to the proximity of fronto-central channels to neural generators involved in sensorimotor processing, such as the sensorimotor cortex, and the supplementary motor area, as well as the auditory system.

Given that entrainment is modulated by the tempo of music, we suggest that complex brain mechanisms might be tuning entrainment, most probably in favor of brain function (Lakatos et al., 2008; Bittman, 2021). The stimulation frequency of 1.65 Hz partially matches the human optimal rate for sensorimotor behavior (Large and Jones, 1999; van Noorden and Moelants, 1999; Drake et al., 2000; McAuley and Jones, 2003). Previous research discussing this match has proposed the existence of a brain mechanism facilitating auditory-motor behavior through entrainment when being presented with auditory stimuli at rates around 2 Hz (Will and Berg, 2007) that could also explain part of our results. However, the likelihood of small unintentional body movements while participants were listening to the music cannot be discounted. Body movement may account for the differences in entrainment between tempos, as such movement might be amplified for the songs at the tempo closer to 2 Hz. Upcoming studies should monitor small body movements, especially head movement, during music listening when exploring entrainment.

To the best of our knowledge, no previous studies had explored how the rate of repetitive auditory stimuli might modulate ASCs’ characteristics. Here, we measured proxies of ASCs both in terms of cognitive function (i.e., reaction time and executive function) and in terms of subjective experience (i.e., by using three subscales of the 11D-ASC) (Studerus et al., 2010) while listening to the music at different tempos. We found that the tempo of electronic music did not affect participants’ overall reaction time or executive capacities. Similarly, participants’ experience of unity and feelings of disembodiment did not change depending on the tempo of the music. These results suggest that the presentation rate of repetitive stimuli does not affect differently these aspects of cognitive function and human experience. Noteworthy, these results do not indicate whether cognition and human experience are altered under the conditions participants where in, as only the rate of stimulation is being accounted for. However, participants felt more experiences of unity for the music at 1.65 Hz compared to the music at 2.85 Hz, mirroring the group-level entrainment findings. While this suggests a possible link between rhythmic stimulation and altered states of consciousness, the absence of a significant individual-level relationship between entrainment and phenomenological experience indicates that additional factors likely contribute to this effect.

One limitation of the study is the potential impact of the course of the experimental procedure on the participants’ phenomenological ratings. Specifically, possible confound effects on the phenomenological experiences reported might rise from having participants conducting the go/no-go task in between listening to the music extracts and filling out the retrospective 11D-ASC questionnaire.

Another potential limitation of the present study is the timing of the reaction-time task, which was performed immediately after the music stopped in all conditions. While participants were instructed to click the mouse as soon as they realized the music had ended, this task required them to divide their attention between the auditory stimuli and the task demands, which may have reduced their full engagement with the music. However, since the task was applied consistently across all conditions, any impact on attention would likely be consistent across conditions as well. Thus, while attention-related factors might have influenced the overall listening experience, the analysis comparing the different conditions should account for these effects. Future research might consider separating the listening experience from task-related demands to better capture the effect of rhythmic auditory stimuli on altered states of consciousness.

Lastly, we acknowledge that the relatively short duration of the rhythmic stimulation epochs, each lasting one minute, may not have provided sufficient time for participants to fully immerse themselves in the music, potentially limiting the strength of the ASC induced. Previous work compared short (i.e., 3 min) versus long (i.e., 15 min) rhythmic and non-rhythmic music stimulation and found that longer durations of rhythmic stimulation led to enhanced auditory thresholds, suggesting that prolonged exposure to rhythmic stimuli may amplify ASC characteristics. Given that no other studies have specifically investigated the impact of stimulation duration on the relationship between entrainment and ASCs, future research should explore the effects of longer rhythmic epochs to further examine how the length of stimulation influences both the depth of entrainment and the intensity of ASCs. This would be especially relevant in light of the fact that entrainment dynamics may vary over time, and longer exposure could potentially result in stronger and clearer connections between neural oscillations and ASC experiences.

In previous literature on ASCs, the usage of repetitive stimulation to trigger altered mental states (Harner, 1990; Krippner, 2000; Walker, 2003; Szabó, 2006; Kjellgren and Eriksson, 2010) has been explained by entrainment (Vaitl et al., 2005), but with no direct evidence to support that claim. Critically, for the first time this study has explored a relationship between the magnitude of entrainment and ASCs. We found three weak, yet significant, brain-behavior associations. Given the modest strength of these relationships, caution should be exercised in interpreting the results and further investigation is warranted to elucidate potential underlying mechanisms. To explore these associations, the neural metric employed was entrainment observed during the final 10 s of stimulation, which was higher for songs at 1.65 Hz compared to both songs at 2.25 Hz and 2.85 Hz.

First, we found that the more differences in entrainment between songs at 1.65 Hz and 2.25 Hz, the more differences in participants’ reaction time to the offset of these songs. In our study, reaction time was used as a measure related to the level of absorption to the music, indicating participants’ cognitive responsiveness and engagement with the auditory stimuli. Because rhythm-induced ASCs are characterized by a selective focus on environmental stimuli (American Psychiatric Association, 1994), reaction time to the offset of the songs is a potential proxy of ASCs. While entrainment was higher for songs at 1.65 Hz compared to songs at 2.25 Hz, the differences in reaction time between these tempos did not exhibit a consistent trend among participants. This is evidenced by the null differences in reaction time across tempos. Also, Figure 7 shows that differences in reaction time between songs at 1.65 Hz and songs at 2.25 Hz are distributed above and below zero. In other words, our results show that higher levels of neural synchronization are related to both faster and slower reaction times, depending on the participant. These findings invite further investigation into the relationship among entrainment, reaction times, and personality traits and/or individual cognitive characteristics. One such significant trait could be musical training, as it was significant in explaining a portion of the observed variance in the strength of entrainment across conditions. However, the limited representation of participants with musical training within our sample precluded a comprehensive investigation into its potential influence on the observed associations.

Second, additional brain-behavior analyses revealed that the greater the differences in entrainment between 1.65 Hz and 2.25 Hz, the more similar participants’ inhibition responses were across these tempo conditions. Finally, the same pattern was found between tempos 1.65 Hz and 2.85 Hz. The inhibition responses were measured as participants’ correct rejections in the go/no-go task performed after listening to the musical extracts. Empirical evidence suggests that the inhibition response relies on neural activity involving the pre-supplementary motor area (Sánchez-Carmona et al., 2019). Also, previous research has shown persistent effects of oscillatory entrainment on cognition (Roberts et al., 2018). In our study, the fronto-central scalp distribution of entrainment observed during listening to the songs suggest that motor areas, including the supplementary motor area, is likely to be recruited. Therefore, it is a possibility that a higher strength of entrainment in motor areas during listening to the songs might decrease neural variability during correct rejection responses in the go/no-go task, resulting in fewer differences in participants’ inhibition response. While this holds physiological interest, its significance in relation to the hypothesized correlation between entrainment and ASCs appears limited. Nevertheless, it does imply that repetitive stimulation effectively entrains regions associated with inhibitory responses, potentially influencing related behavioral outcomes. Consequently, future investigations should delve into the potential correlation between entrainment, inhibitory responses, and ASCs.

A promising direction for future research would be to investigate parallels between rhythm-induced ASCs and the effects of psychedelics on alpha-band activity in the default mode network (DMN). Psychedelics are known to impair self-referential processing, often resulting in decreased alpha-band activity and altered connectivity within the DMN, particularly in regions such as the medial prefrontal cortex and posterior cingulate cortex (Gattuso et al., 2023). A similar pattern may be observed in rhythm-induced ASCs, where repetitive auditory stimuli could entrain neural activity in fronto-central regions, potentially disrupting DMN functioning. To test this hypothesis, future studies could examine whether rhythmic auditory stimuli lead to entrainment in fronto-central regions and a reduction in alpha-band activity in the DMN, thereby suggesting altered DMN functioning. These effects should be compared to those induced by control stimuli, such as non-rhythmic auditory patterns, to better isolate the specific role of rhythmic stimulation in modulating DMN activity. In the present study, we focused on fronto-central regions, as these demonstrated the highest levels of entrainment. However, future studies could extend the analysis to other brain regions, such as the posterior cingulate cortex (PCC), which may also play a significant role in modulating self-referential processes during ASCs.

To establish neural entrainment as a causal mechanism underlying rhythm-induced ASCs, future research should incorporate methodologies that allow for direct manipulation of neural oscillations. One promising approach is transcranial magnetic stimulation (TMS), which can be used to selectively entrain neural rhythms at specific frequencies (Thut et al., 2011; Rahnev, 2013; Corlier et al., 2023) and assess their impact on ASC-related experiences. This approach would allow for a more precise measurement of entrainment by eliminating the spectral complexity inherent in music stimuli, as well as the confounding effects of emotional responses and participants’ individual musical experience. Simultaneous EEG-TMS could help elucidate the causal role of entrainment by measuring how artificial modulation of neural rhythms and its strength influences both subjective experiences and cognitive performance.

However, an ecological approach remains equally valuable. For instance, future studies could incorporate mobile EEG in “pop-up” festivals featuring electronic music in real-world settings, allowing for a more naturalistic interaction between individuals and the music while still maintaining some level of control over experimental conditions.

Lastly, incorporating control conditions such as non-rhythmic auditory stimuli or silence is crucial to isolate the effects of rhythmic stimulation on ASCs. Silence serves as a true control condition by virtue of its absence of auditory stimulation and thus should not be disregarded, as it allows for a baseline comparison of the neural and phenomenological effects of rhythmic versus non-rhythmic stimuli. By combining these approaches, future research can move beyond correlational findings to establish a direct causal link between neural entrainment and ASCs, shedding light on the fundamental mechanisms behind rhythm-induced altered states.

## 5 Conclusion

Previous research on ASCs attributes the effects of repetitive stimulation on altered mental states to entrainment, though without direct evidence. This is relevant in electronic dance music events, where the strong rhythmic beats—akin to drumming—may play a key role in inducing ASCs and fostering feelings of connectedness and transcendence. To our knowledge, this is the first systematic study demonstrating a relationship between entrainment and ASCs. In summary, this article has argued that entrainment and the phenomenological aspects of ASCs induced by repetitive stimuli are related. Our results showing that entrainment is higher for stimulation rates at 1.65 Hz are broadly consistent with previous findings. We found an association between entrainment and absorption to the music, as measured with participants’ reaction times to the offset of the stimulation. Specifically, we observed that the more the strength of participants’ entrainment to the music, as measured by the differences in entrainment between songs at 1.65 Hz and 2.25 Hz, the more differences in participants’ reaction time to the offset of these songs. While entrainment was higher for songs at 1.65 Hz compared to 2.25 Hz, reaction time was the same across tempos. Therefore, we suggest that individual personality or cognitive traits might be modulating whether the strength of entrainment is related to more or less time to respond and, subsequently, to whether participants are more or less absorbed by the songs. Although musical training emerged as a significant factor explaining variance in the magnitude of entrainment, the small subset of participants with musical training in our sample limits deeper exploration of its impact on this brain-behavior association. Additionally, given the weak strength of this relationship, caution is advised when interpreting these findings.

## Data Availability

The datasets presented in this study can be found in online repositories. The names of the repository/repositories and accession number(s) can be found at: https://osf.io/nkzcg/.
