# Supplementary material for: The strength of neural entrainment to electronic music correlates with proxies of altered states of consciousness
Source: Front Hum Neurosci. 2025 Apr 9;19:1574836. doi: 10.3389/fnhum.2025.1574836 (PMC12014595; doi:10.3389/fnhum.2025.1574836)
Supplement: Supplementary file 1 [file Data_Sheet_1.docx]

Supplementary Material

# Supplementary material A1: Linear mixed model specifications in Wilkinson-Rogers notation for assessing changes in global entrainment across tempos

A linear mixed model analysis was conducted to explore the effect of the tempo of the music on global entrainment. Significant reductions in the Bayesian information criterion when adding variables to the null regression model were used to identify the model that best fits the data. The null model included only the intercept (1). The model with the best fit included tempo as a dummy variable (three levels: Tempo1.25, Tempo2.25, and Tempo2.85; Tempo1.25 is taken as the reference), the covariates "musical training" and "years of musical training", and each participant’s identification code as a random effect (2).

| $Global entrainment \sim1+(1\vert Participant ID)$ | (1) |
| --- | --- |
| $Global entrainment \sim Tempo2.25+Tempo2.85+Musical training+Years of musical training+(1\vert Participant ID)$ | (2) |

# Supplementary material A2: Linear model specifications in Wilkinson-Rogers notation for assessing brain-behavior relationships

Linear regression models were used to explore whether the magnitude of entrainment is related to participant’s behavioral measures (i.e., reaction time, executive function, Experience of unity, Spiritual experience, and Disembodiment). A separate linear model was fitted for each neural and behavioral difference score computed between pairs of tempos (3–5). Computing difference scores aimed to uncover whether states in which the brain is highly synchronized to the beats of the songs vs. less synchronized is related to the variability in the participant’s performance between the two conditions. e measure of entrainment included in the models differed between objective and subjective behavioral measures. For objective measures (i.e., reaction time and executive function), entrainment during the last 10 seconds of stimulation was used, whereas for subjective measures (i.e., experience of unity, spiritual experience, and disembodiment), global entrainment was used.

| $Behavioral Measure \left( 1.65-2.25 \right) \sim Entrainment (1.62-2.25)$ | (3) |
| --- | --- |
| $Behavioral Measure \left( 1.65-2.85 \right) \sim Entrainment(1.62-2.85)$ | (4) |
| $Behavioral Measure \left( 2.25-2.85 \right) \sim Entrainment(2.25-2.85)$ | (5) |
